# Butyrate Supplementation at High Concentrations Alters Enteric Bacterial Communities and Reduces Intestinal Inflammation in Mice Infected with *Citrobacter rodentium*

**DOI:** 10.1128/mSphere.00243-17

**Published:** 2017-08-23

**Authors:** Janelle A. Jiminez, Trina C. Uwiera, D. Wade Abbott, Richard R. E. Uwiera, G. Douglas Inglis

**Affiliations:** a Agriculture and Agri-Food Canada, Lethbridge, Canada; b Department of Agricultural, Food and Nutritional Science, University of Alberta, Edmonton, Canada; c Department of Surgery, Faculty of Medicine and Dentistry, University of Alberta, Edmonton, Canada; University of Wisconsin-Madison

**Keywords:** butyrate, *Citrobacter rodentium*, inflammation, intestine, mice, microbiota

## Abstract

Butyrate is a short-chain fatty acid by-product of the microbial fermentation of dietary fermentable materials in the large intestine; it is the main energy source for enterocyte regeneration, modulates the enteric microbial community, and contributes to increasing host health via mechanisms that are relatively poorly defined. Limited research has examined the therapeutic potential of butyrate using models of enteric inflammation incited by pathogenic organisms. We used *Citrobacter rodentium* to incite acute Th1/Th17 inflammation to ascertain the impact of butyrate on the host-microbiota relationship. Rectal administration of 140 mM butyrate to mice increased fecal concentrations of butyrate and increased food consumption and weight gain in mice infected with *C. rodentium*. Histological scores of colonic inflammation were lower in infected mice administered 140 mM butyrate. Expression of *Il10*, *Tgfβ*, and *Muc2* was elevated in noninfected mice administered butyrate in comparison to mice not administered butyrate. Infected mice administered butyrate displayed elevated expression of genes necessary for pathogen clearance (i.e., *Il17A* and *Il1β*) and of genes involved in epithelial barrier repair and restoration (i.e., *Relmβ*, *Tff3*, and *Myd88*). Butyrate supplemented to inflamed colons increased the abundances of *Proteobacteria* and *Lachnospiraceae* and reduced the abundance of *Clostridiaceae* species. Mice with enteritis that were administered butyrate also exhibited an increased abundance of mucus-associated bacteria. In summary, rectal administration of butyrate increased feed consumption and weight gain, ameliorated *C. rodentium*-induced cell injury through enhanced expression of immune regulation and tissue repair mechanisms, and increased the abundance of butyrate-producing bacteria in mice with enteritis.

**IMPORTANCE** The study findings provide evidence that administration of butyrate in a dose-dependent manner can increase weight gain in infected mice, enhance clearance of the infection, reduce inflammation through altered cytokine expression, and enhance tissue repair and mucus secretion. Moreover, butyrate treatment also affected the abundance of bacterial populations in both noninflamed and inflamed intestines. Notably, this investigation provides foundational information that can be used to determine the effects of prebiotics and other functional foods on the production of butyrate by enteric bacteria and their impact on intestinal health and host well-being.

## Abstract (precis)

The study findings provide evidence that administration of butyrate in a dose-dependent manner can improve the weight gain of infected mice, enhance clearance of the infection, reduce inflammation through altered cytokine expression, and enhance tissue repair and mucus secretion. Moreover, butyrate treatment also affected the abundance of bacterial populations in both noninflamed and inflamed intestines. Notably, this investigation provides foundational information that can be used to determine the effects of prebiotics and other functional foods on the production of butyrate by enteric bacteria and their impact on intestinal health and host well-being.

## INTRODUCTION

Butyrate is a short-chain fatty acid (SCFA) that is produced by the fermentation of dietary fiber in the large intestine and is purported to confer a vareity of health benefits. The role of butyrate is to provide energy to colonocytes, as it is the preferred energy source compared to other SCFAs produced within the colon (1). In people, concentrations of colonic butyrate can range from 10 to 20 mM (2), and approximately 95% to 99% of all SCFAs produced in the colon are rapidly absorbed and metabolized into energy sources by colonic cells (3). A failure to utilize butyrate as an energy source can enhance the symptoms of intestinal disease. As an example, individuals with ulcerative colitis often have metabolic deficiencies in butyrate transport systems, and often these systems play a role in butyrate absorption and usage, suggesting a reduced ability to absorb butyrate (4). The optimal concentration of butyrate within the host has long been disputed. Studies that use experimental models of colitis in rodents have reported administering butyrate at concentrations ranging from 40 mM to 130 mM (5–9). Furthermore, concentrations of butyrate administered to people with ulcerative colitis via enemas have ranged from 80 mM to 150 mM (10). A limited number of researchers have investigated changes in mucus secretion resulting from butyrate enemas in animal models or have administered butyrate at concentrations as high as 100 mM in their models. Therefore, the high absorptive rate of intestinal SCFA necessitates that higher concentrations be used in scientific investigations (6, 11).

The addition of butyrate directly to human colons (12), colonic epithelial cells (13), and carcinoma epithelial cells (14) has been shown to be beneficial in reducing intestinal inflammation (15–17). *In vitro* analyses have shown that butyrate can reduce expression of proinflammatory cytokines and enhance epithelial barrier function, and *in vivo* studies conducted in mice without enteritis suggest that butyrate increases mucus synthesis in goblet cells (6, 10). Furthermore, several studies using intestinal cell lines have shown that the administration of butyrate to intestinal cells can downregulate NF-κB and reduce the expression of proinflammatory cytokines (18–20), and this has also been observed in mouse models of chemically induced enteritis (21, 22). The degree and mechanisms by which butyrate influences pathogen-induced inflammation, including its impact on dysbiosis and intestinal injury, have not be extensively studied in animal models.

Some evidence indicates that butyrate plays a role in maintaining the intestinal barrier by increasing the expression of mucins such as MUC2 and enhancing mucus production (23). Intestinal mucins are composed of transmembrane or secretory glycoproteins released from goblet cells and are involved in forming two protective mucus layers, with MUC2 being the major secretory glycoprotein in the colon (24, 25). Although the mucus layer is thought to inhibit bacterial binding to the epithelium, thereby limiting bacterial entry into the gut-associated lymphoid tissue (GALT) and the subsequent induction of proinflammatory responses (26), there are bacterial species that utilize mucus as an energy source, readily colonize mucus layers, and act as indicators of mucus abundance. *Akkermansia muciniphila* is a well-known mucus-degrading organism and has often been isolated from the colons of mice and people (27), whereas *Mucispirillum schaedleri* has been shown to selectively colonize intestinal mucus in a variety of organisms (28). More recently, studies investigating the effect of butyrogenic bacteria on the intestinal mucus barrier showed that changes in intestinal bacterial populations influenced the production and secretion of mucins from goblet cells (29). Moreover, treatment with butyrate also altered bacterial populations of *Bacteroidetes*, *Firmicutes*, *Deferribacteres*, and *Proteobacteria* in the large intestine (30–33). We hypothesized that butyrate supplemented to acutely inflamed colons would contribute to a temporal and spatial increase in mucus secretion and would concomitantly reduce proinflammatory signaling and inflammation. To test this hypothesis, we incited acute inflammation with *Citrobacter rodentium* (with or without rectally administered butyrate) and temporally measured a variety of variables, including food consumption and weight gain, intestinal SCFA concentrations, colonic cell damage and injury, the expression of genes involved in proinflammatory immune responses and repair, and the overall changes in the colonic bacterial community structure.

## RESULTS

### *Citrobacter rodentium* incited enteritis in C57BL/6 mice.

**FIG 1:**
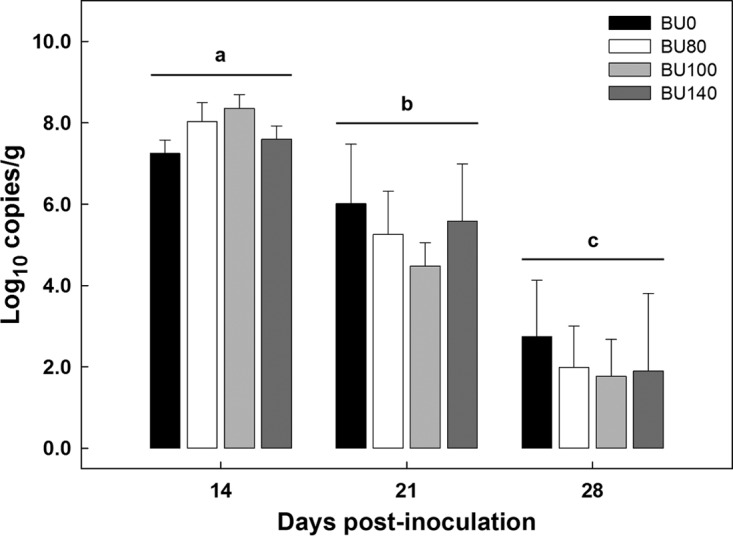
Densities of *C. rodentium* in feces from mice inoculated with the bacterium and rectally administered PBS (BU0) or butyrate at a concentration of 80 mM (BU80), 100 mM (BU100), or 140 mM (BU140) from the point of inoculation to day 28 postinoculation. No *C. rodentium* was detected in feces from mice not inoculated with the bacterium. Vertical lines associated with bars represent standard errors of the means (*n* = 4). There was no difference (*P* = 0.965) in the densities of *C. rodentium* among the butyrate treatments, but densities differed (*P* < 0.001) among the three sample times. Time groups not indicated by the same letter differ (*P* ≤ 0.012).

**FIG 2:**
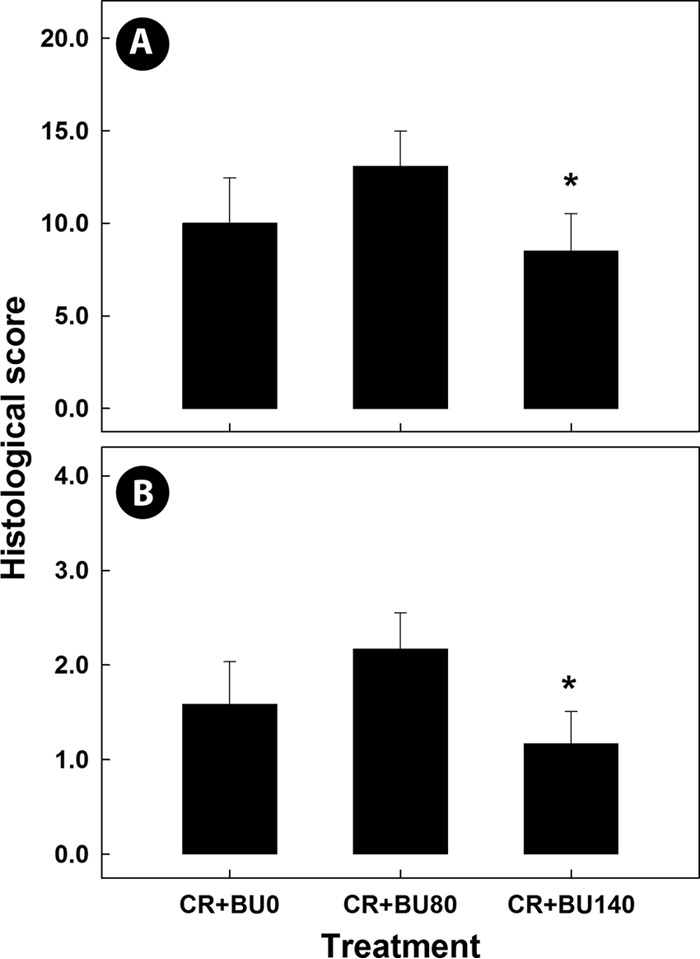
Histopathologic changes in colonic tissue from mice inoculated with *C. rodentium* (CR) and rectally administered PBS (BU0) or butyrate at concentrations of 80 mM (BU80) and 140 mM (BU140) averaged over peak and late infection. (A) Total histopathologic scores. (B) Epithelial cell hyperplasia. Vertical lines associated with histogram bars represent standard errors of the means (*n* = 4). *, *P* < 0.050 (relative to the BU0 treatment). For all treatments, histopathologic changes were reduced over time (*P* ≤ 0.005) in mice infected with *C. rodentium*.

To confirm the presence of *C. rodentium* in mice exhibiting symptoms of enteritis and to ascertain whether the butyrate concentration affected colonization by the bacterium, fecal samples were collected and densities of the bacterium were quantified. In control mice administered phosphate-buffered saline (PBS) via gavage, *C. rodentium* was not isolated from fecal samples. In contrast, all mice with enteritis shed the bacterium in their feces (Fig. 1; see also Fig. S1 in the supplemental material). Butyrate administration had no impact (*P* = 0.965) on temporal densities of *C. rodentium* in feces; shedding of *C. rodentium* peaked on day 14 postinoculation (p.i.), and densities of the bacterium decreased (*P* < 0.050) thereafter. To confirm that mice administered *C. rodentium* via gavage exhibited phenotypic signs of enteritis, colonic tissues were examined histopathologically. Mice that were not administered *C. rodentium* via gavage did not show symptoms of infection, exhibit overt evidence of enteric epithelial cell hyperplasia or epithelial cell injury, or show changes in mitotic activity, goblet cell presence, or crypt height, with total average histopathologic scores of less than 1.30 ± 0.25. In contrast, all mice administered *C. rodentium* via gavage presented symptoms and signs of enteritis and exhibited substantially higher (*P* ≤ 0.001) histopathologic scores (Fig. 2) than mice without enteritis (data not presented) for all of the categories examined. The degree of tissue injury was highest on day 14 p.i. (i.e., peak infection), decreased by day 21 (i.e., late infection), and was minimal by day 28 (i.e., clearance).

10.1128/mSphere.00243-17.1 FIG S1  Densities of *C. rodentium* in feces from mice inoculated with the bacterium and rectally administered PBS (BU0), or butyrate at a concentration of 80 mM (BU80), or 140 mM (BU140) from the point of inoculation to day 28 postinoculation. No *C. rodentium* was isolated from feces of mice that were not inoculated with the bacterium. Download FIG S1, PDF file, 0.1 MB. © Crown copyright 2017. 2017 Crown This content is distributed under the terms of the Creative Commons Attribution 4.0 International license.

### Fecal butyrate concentrations were higher in infected mice administered butyrate rectally.

**FIG 3:**
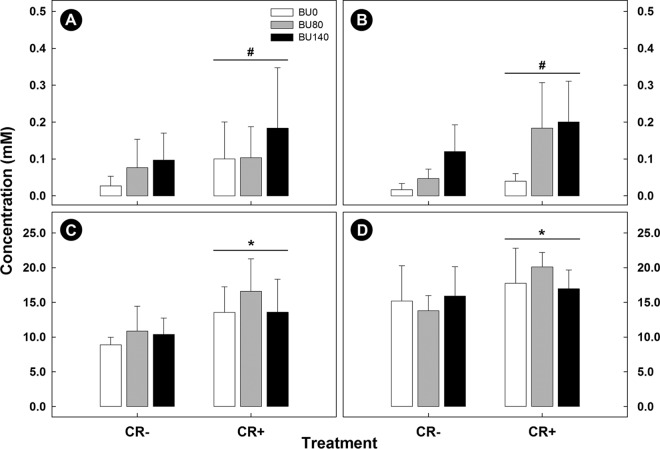
Concentrations of butyrate and total short-chain fatty acids (SCFA) measured in feces from mice inoculated with PBS (CR−) or *C. rodentium* (CR+) via gavage and rectally administered PBS (BU0) or butyrate at concentrations of 80 mM (BU80) and 140 mM (BU140). Samples were collected on days 14 and 21 postinoculation (p.i.). (A) Butyrate concentrations in feces collected on day 14 p.i. (B) Butyrate concentrations in feces collected on day 21 p.i. (C) Total SCFA concentrations in feces collected on day 14 p.i. (D) Total SCFA concentrations in feces collected on day 21 p.i. Vertical lines associated with histogram bars represent standard errors of the means (*n* = 4). #, *P* ≤ 0.073 (between infected and noninfected mice). *, *P* ≤ 0.020 (between infected and noninfected mice).

To determine whether rectal butyrate administration altered fecal SCFA concentrations, fresh fecal samples were collected and analyzed for individual SCFAs. There was no effect (*P* = 0.56) of time p.i. on the overall concentration of butyrate measured in feces (Fig. 3A and B). However, mice with enteritis exhibited a trend for higher (*P* ≤ 0.073) quantities of butyrate in their feces than mice without enteritis, and infected mice that were administered butyrate had higher (*P* < 0.050) concentrations of butyrate in their feces than mice administered PBS rectally. Total SCFA concentrations measured in feces were also higher (*P* ≤ 0.020) in mice inoculated with *C. rodentium* than in mice administered PBS via oral gavage (Fig. 3C and D).

### Butyrate supplementation increased weight gain and feed consumption in mice with enteritis during peak infection.

**FIG 4:**
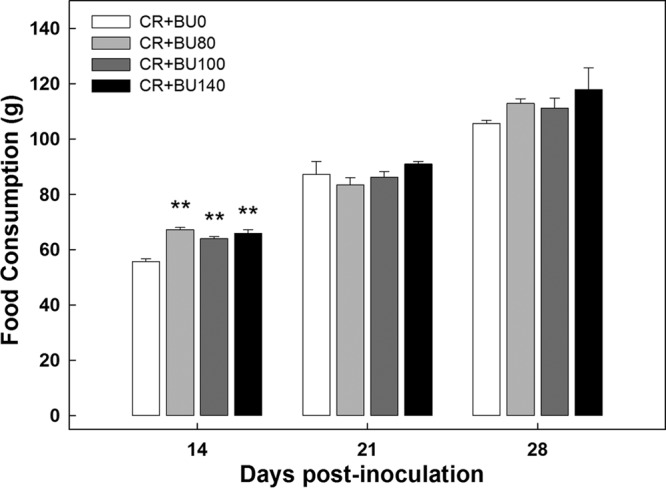
Daily food consumption in mice inoculated with *C. rodentium* (CR+) and rectally administered PBS (BU0) or butyrate at concentrations of 80 mM (BU80), 100 mM (BU100), and 140 mM (BU140). Vertical lines associated with markers represent standard errors of the means (*n* = 4). **, *P* ≤ 0.010 (relative to the BU0 treatment on day 14 postinoculation).

**FIG 5:**
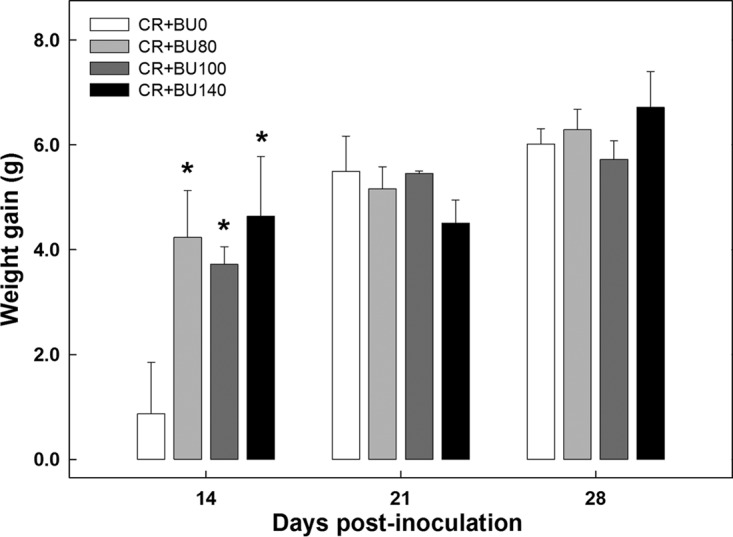
Weight gain in mice inoculated with *C. rodentium* (CR+) and rectally administered PBS (BU0) or butyrate at concentrations of 80 mM (BU80), 100 mM (BU100), and 140 mM (BU140). Vertical lines associated with histogram bars represent standard errors of the means (*n* = 4). *, *P* ≤ 0.050 (relative to the BU0 treatment).

In order to measure whether the rectal administration of butyrate had a physiological effect on the mouse during *C. rodentium* infection, average feed consumption and weight gain values were analyzed. Butyrate administration did not affect feed consumption (*P* > 0.95) or weight gain (*P* > 0.44) in mice without enteritis at any of the three sample times (data not presented). At peak infection, mice with enteritis that had been administered butyrate consumed more (*P* = 0.003) food than mice with enteritis that had not been administered butyrate (Fig. 4). During late infection (*P* = 0.38) and clearance (*P* = 0.34), there was no difference in food consumption between control mice and those supplemented with butyrate. Mice with *C. rodentium*-induced enteritis that were administered butyrate gained weight faster (*P* ≤ 0.002) than mice not administered butyrate at peak infection (Fig. 5) but not at late infection or clearance (*P* ≥ 0.53) (data not presented).

### High butyrate concentrations decreased epithelial cell hyperplasia in mice with enteritis.

To evaluate a potential decrease in distal colonic inflammation due to butyrate supplementation, representative samples of distal colonic tissue were collected and analyzed for histopathologic changes. Mice with enteritis exhibited total histopathologic scores of ≥14.0 ± 2.7 at peak infection, ≥9.3 ± 3.5 at late infection, and ≤6.5 ± 0.1 at clearance (Fig. 2A). Scores of total histopathologic changes did not differ (*P* = 0.19) between peak and late infection but were lower (*P* ≤ 0.009) at clearance. Averaged over time, total pathological changes (*P* = 0.020) and epithelial cell hyperplasia levels (*P* = 0.023) were lower in mice with enteritis that had been administered butyrate at a concentration of 140 mM (Fig. 2) than in mice not administered butyrate. Increased (*P* = 0.054) mitotic activity was also observed in mice with enteritis that had been administered butyrate at 80 mM at peak infection compared to mice with enteritis not administered butyrate (Fig. S2).

10.1128/mSphere.00243-17.2 FIG S2  Mitotic cell activity in mice inoculated with *C. rodentium* (CR+) and rectally administered PBS (BU0), or butyrate at a concentration of 80 mM (BU80), or 140 mM (BU140). Vertical lines associated with histogram bars represent standard errors of the means (*n* = 4). *, *P* ≤ 0.050 (relative to the BU0 treatment averaged over the indicated time points). In all instances, infection with *C. rodentium* affected mitotic cell activity over time (*P* ≤ 0.005). Download FIG S2, PDF file, 0.1 MB. © Crown copyright 2017. 2017 Crown This content is distributed under the terms of the Creative Commons Attribution 4.0 International license.

### Mucus accumulated in the lumen after butyrate supplementation at peak infection.

**FIG 6:**
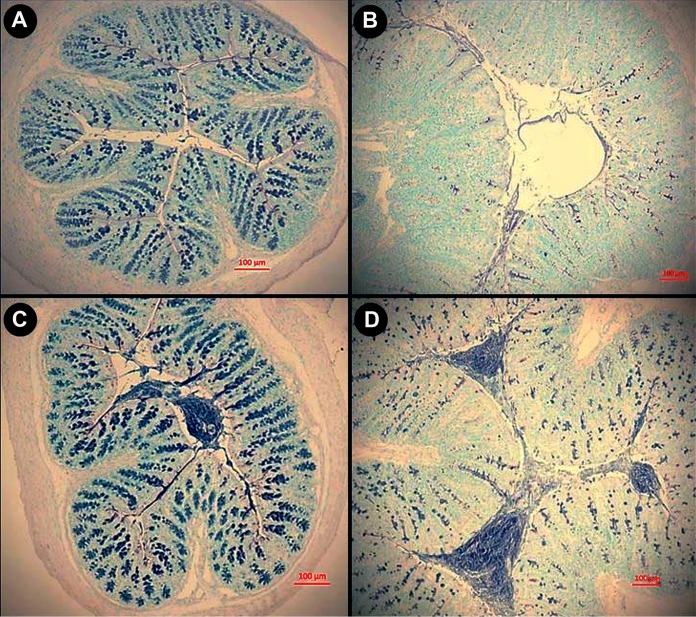
Mucus localization in alcian blue–periodic acid-Schiff-stained sections from the distal colons of mice inoculated with PBS (CR−) or *C. rodentium* (CR+) and rectally administered PBS (BU0) or butyrate at a concentration of 140 mM (BU140) at day 14 p.i. (A) CR–, BU0. (B) CR+, BU0. (C) CR–, BU140. (D) CR+, BU140. Tissue from infected mice administered butyrate exhibited a consistent increase in the density of mucus (blue stain) within goblet cells and the colonic lumen. Bars, 100 µm.

To confirm the hypothesis that butyrate supplementation affects mucus accumulation in the colon, representative tissues from the distal colon were stained for mucus presence. At peak and late infection, mice with enteritis that had been administered butyrate at a concentration of 140 mM exhibited increased accumulation of mucus in the colonic lumen and within goblet cells relative to mice administered PBS enemas (Fig. 6; Fig. S3).

10.1128/mSphere.00243-17.3 FIG S3  Mucus localization in alcian blue–periodic acid-Schiff-stained sections of the distal colons of mice inoculated with PBS (CR−) or *C. rodentium* (CR+), and rectally administered PBS (BU0) or butyrate at a concentration of 140 mM (BU140) at day 21 p.i. (A) CR−, BU0. (B) CR+, BU0. (C) CR−, BU140. (D) CR+, BU+. Tissue from infected mice supplemented with butyrate exhibited an increase in the density of mucus (blue stain) within the colonic lumen. Overall, more mucus was observed in the lumen on day 21 p.i. than on day 14 p.i. Bar, 100 μm. Download FIG S3, PDF file, 0.6 MB. © Crown copyright 2017. 2017 Crown This content is distributed under the terms of the Creative Commons Attribution 4.0 International license.

### Butyrate supplementation affected enteric gene expression in mice with enteritis.

**FIG 7:**
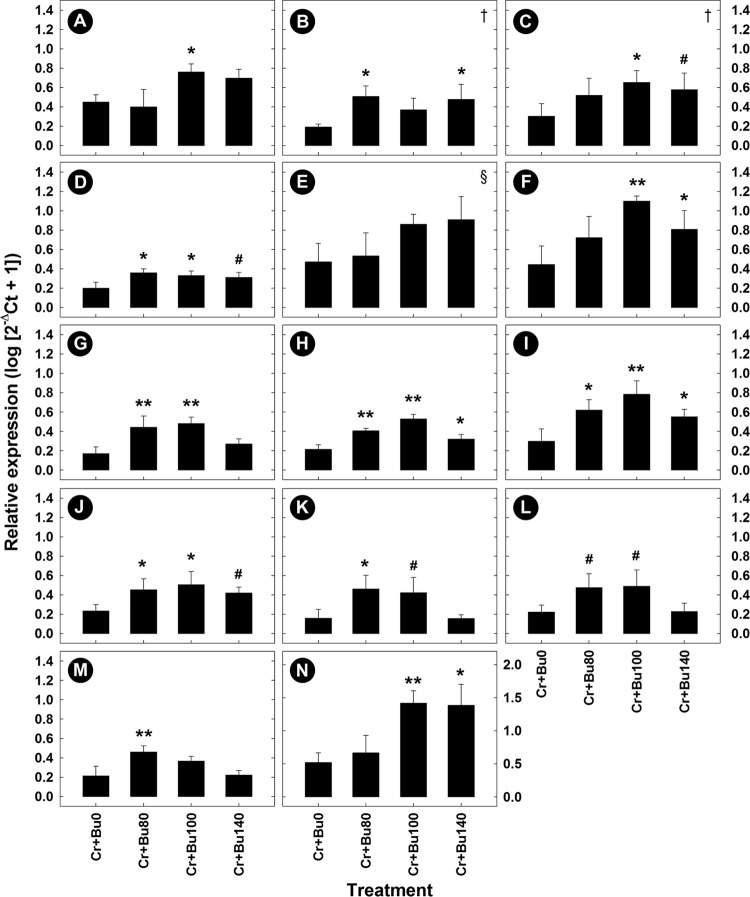
Relative mRNA gene expression profiles of cytokine-related innate barrier function and host pathogen recognition genes measured in colonic tissue harvested from mice inoculated with *C. rodentium* (CR) and with rectally administered PBS (BU0) or butyrate at concentrations of 80 mM (BU80), 100 mM (BU100), and 140 mM (BU140) averaged over time. (A) *Il17A*. (B) *Il22*. (C) *Il1β*. (D) *MyD88*. (E) *RegIIIγ*. (F) *Ifnγ*. (G) *Tlr9*. (H) *Tlr2*. (I) *Tnfα*. (J) *Ltb4R1*. (K) *Prg3*. (L) *Il4*. (M) *Tff3*. (N) *Relmβ*. Vertical lines associated with histogram bars represent standard errors of the means (*n* = 4). #, *P* ≤ 0.100; *, *P* ≤ 0.050; **, *P* ≤ 0.010 (relative to the BU0 treatment). †, the statistical value represents a difference determined by excluding a butyrate treatment and comparing CR+ BU0 treatment to only two of the three other treatments; §, the statistical value represents comparison between CR+ BU0 treatment and CR+ BU100 treatment only. Ct, threshold cycle.

**FIG 8:**
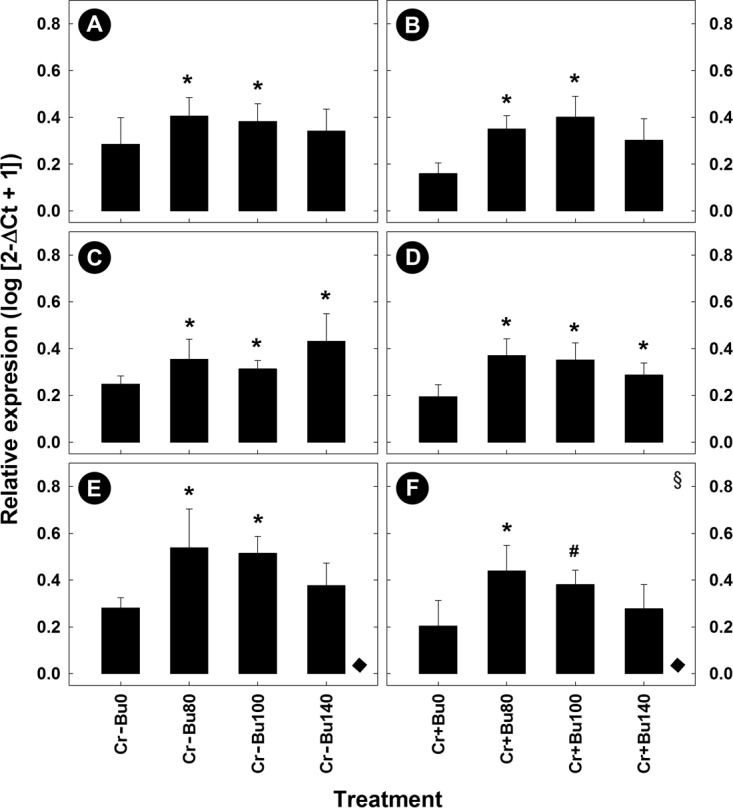
Relative mRNA gene expression profiles of regulatory cytokines and mucus-producing genes in colonic tissue harvested from mice inoculated with PBS (CR−) or *C. rodentium* (CR+) and rectally administered PBS (BU0) or butyrate at concentrations of 80 mM (BU80), 100 mM (BU100), and 140 mM (BU140). (A and B) *Il10*. (C and D) *Tgfβ*. (E and F) *Muc2*. Vertical lines associated with histogram bars represent standard errors of the means (*n* = 4). #, *P* ≤ 0.100; *, *P* ≤ 0.050 (relative to the BU0 treatment). ♦, *P* = 0.053 (representing the difference in overall *Muc2* expression due to *C. rodentium* infection). §, the statistical value represents a difference determined by excluding a butyrate treatment and comparing CR+ BU0 treatment to only two of the three other treatments.

Representative samples from the distal colon were collected and RNA was extracted from them to determine the degree to which butyrate administration influenced mRNA expression of genes related to infection and inflammation. The administration of butyrate had no effect (*P* ≥ 0.16) on expression of Th1 (*Ifnγ*, *Tnfα*)-, Th2 (Il4)-, and Th17 (*Il17A*, *Il22*)-associated cytokines or on expression of *Myd88*, *Prg3*, *RegIIIγ*, *Tff3*, and *Tlr9* in mice without enteritis relative to mice that were not administered butyrate (data not presented). In contrast, mice administered butyrate at a concentration of 80 mM exhibited a trend of increased (*P* = 0.062) expression of Th2 (*Il4*) cytokines and of significantly increased (*P* ≤ 0.050) expression of Th1 (*Ifnγ*, *Tnfα*) and Th17 (*Il22*) cytokines in mice with enteritis (Fig. 7); mice administered butyrate at concentrations of 100 mM and higher also exhibited increased (*P* ≤ 0.050) expression of *Il1β*. The administration of butyrate at 80, 100, and 140 mM to mice with enteritis increased (*P* ≤ 0.036) the expression of genes involved in bacterial recognition and defense, including *Myd88*, *Tlr2*, and *Ltb4r1*, but only the mice given butyrate at 80 mM (*P* = 0.005) and 100 mM (*P* = 0.004) exhibited increased expression of *Tlr9*. Mice given butyrate at 80 mM (*P* = 0.044) and 100 mM (*P* = 0.076) but not 140 mM (*P* = 0.990) exhibited increased expression of *Prg3*. Butyrate given at 100 mM also increased (*P* = 0.028) expression of *RegIIIγ*, and administration of 100 mM (*P* = 0.007) and 140 mM (*P* = 0.012) butyrate increased expression of *Relmβ*, while butyrate provided at 80 mM (*P* = 0.002) and 100 mM increased (*P* = 0.045) expression of *Tff3* in mice with enteritis. There was no difference (*P* ≥ 0.36) between the PBS and *C. rodentium* treatments in the expression of the Treg response cytokines *Il10* and *Tgfβ* in mice not administered butyrate (Fig. 8A to D). In mice administered butyrate at 80 and 100 mM, expression of *Il10* was increased (*P* ≤ 0.028), and all concentrations of butyrate stimulated an increase (*P* = 0.049) in *Tgfβ* expression in mice with and without enteritis (Fig. 8A to D). Regardless of butyrate administration, mice with enteritis displayed a trend of decreased (*P* = 0.053) *Muc2* expression relative to mice without enteritis. Administration of butyrate at concentrations of 80 and 100 mM increased *Muc2* expression in mice without enteritis (*P* < 0.023) and demonstrated a trend for increased (*P* < 0.088) *Muc2* expression in mice with enteritis (Fig. 8E and F).

### Butyrate supplementation reduced the abundance of *Firmicutes* and butyrate-producing bacteria in the distal colon.

**FIG 9:**
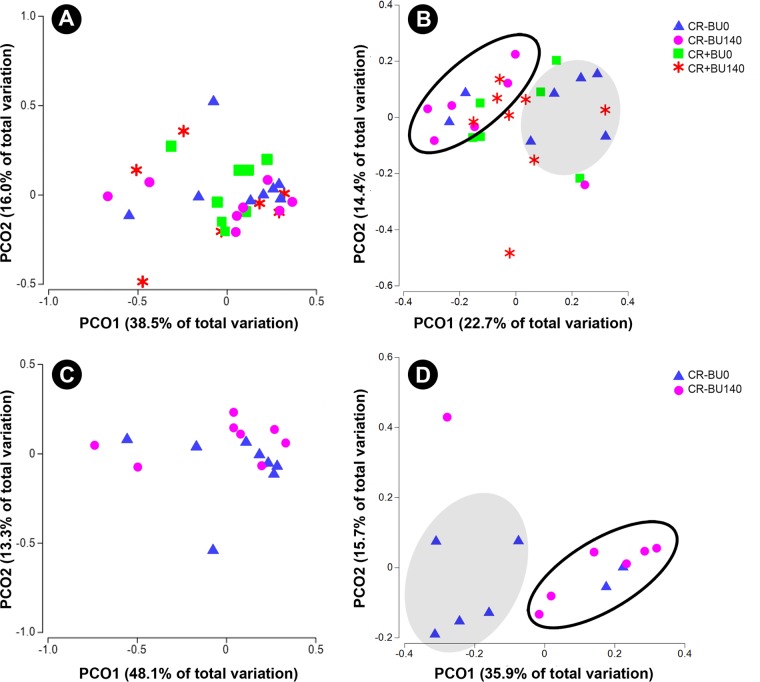
Effects of rectal administration of PBS (BU0) or 140 mM butyrate (BU140) on bacterial community structures in the distal colon of mice (mucosa-associated and within digesta) inoculated with PBS (CR−) or *C. rodentium* (CR+) as determined by weighted UniFrac analysis. Axes identify percent variation among treatments, and ellipsoids are used to highlight clustering of communities by treatment. (A and B) Mice with (CR+) and without (CR−) enteritis. (C and D) Mice without enteritis (CR−). (A) Mucosa-associated. (B) Digesta. The shaded ellipsoid highlights clustering of communities from CR− BU0 treatment mice, and the open ellipsoid highlights clustering of communities from CR− BU140 treatment mice; a butyrate effect was observed for the CR− treatments (*P* = 0.071 with 753 random permutations). (C) Mucosa-associated. (D) Digesta. The shaded ellipsoid highlights clustering of communities from CR− BU0 treatment mice, and the open ellipsoid highlights clustering of communities from CR− BU140 treatment mice; a butyrate effect was observed (*P* = 0.071 with 762 random permutations).

**FIG 10:**
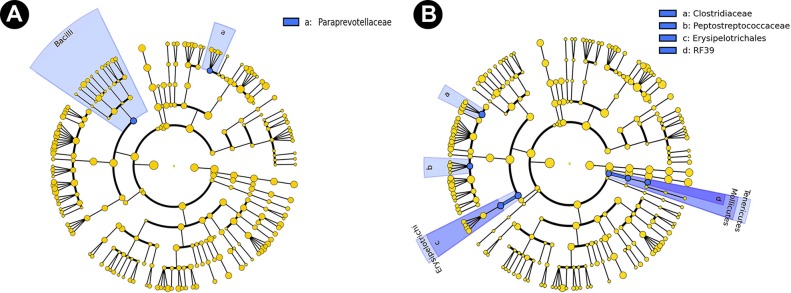
Phylogenetic trees of bacteria associated with mucosa and within digesta in the distal colons of mice with (CR+) and without (CR−) enteritis and rectally administered PBS (BU0) or 140 mM butyrate (BU140) as determined using the linear discriminant analysis effect size (LEfSe) method (LDA value, >2.0). The abundances of bacterial taxa highlighted in blue differed (*P* ≤ 0.050) between the BU0 and BU140 treatments. (A) Mucosa-associated. (B) Digesta. Data to construct the phylogenetic trees used summarized taxonomic values per treatment averaged over four replications.

**FIG 11:**
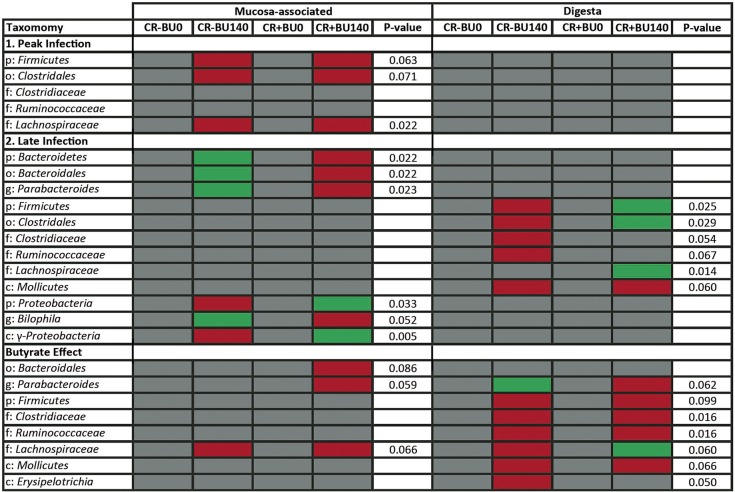
Changes observed in mucosa- and digesta-associated bacterial communities within the distal colon at peak infection and late infection, including the overall butyrate effect. Green boxes indicate a significant (*P* ≤ 0.100) increase in the abundance of specified taxa supplemented with butyrate (BU140) compared to the PBS control (BU0) within each enteritis treatment group. Red boxes indicate a significant (*P* ≤ 0.100) decrease in the abundance of specified taxa supplemented with butyrate (BU140) compared to butyrate control taxa (BU0) within each enteritis treatment group. p, phylum; c, class; o, order; f, family; g, genus. A butyrate effect was defined as a significant increase or decrease in bacterial abundance in response to butyrate administration averaged over time.

**FIG 12:**
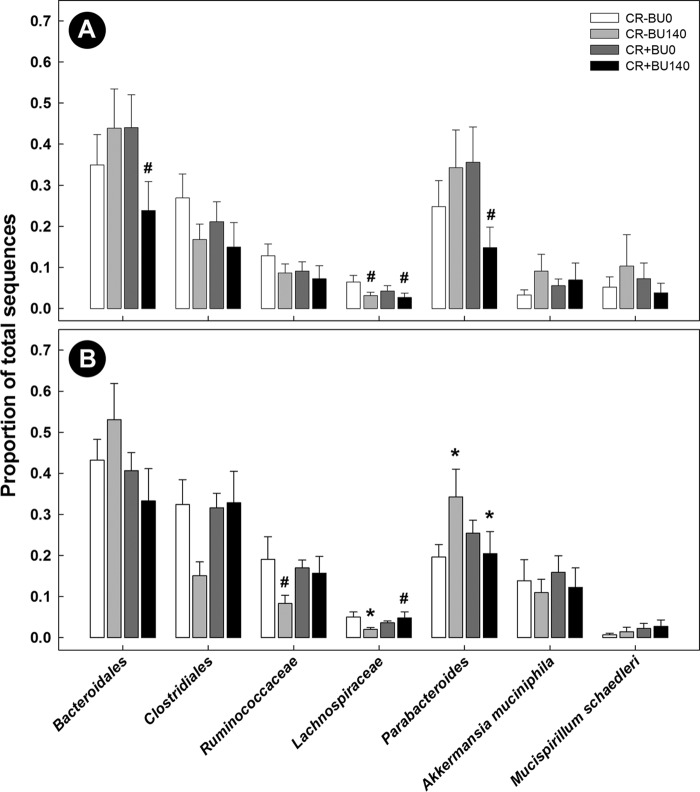
Abundance of bacteria within the distal colon of mice inoculated with PBS (CR−) or *C. rodentium* (CR+) and rectally administered PBS (BU0) or butyrate at a concentration of 140 mM (BU140). (A) Mucosa-associated. (B) Digesta. Vertical lines associated with histogram bars represent standard errors of the means (*n* = 4). #, *P* ≤ 0.100; *, *P* ≤ 0.050 (comparing butyrate treatment effects within CR− mice [BU0 to BU140] and CR+ mice [BU0 to BU140]).

To determine the changes in bacterial community structure due to butyrate supplementation, representative samples from the distal colon (mucosal and digesta) of all mice were collected and community DNA was sequenced for individual animals (i.e., to obtain a measure of variability among replicate mice). Based on UniFrac analyses of community similarities, the structure of the mucosa-associated bacterial community was more variable and differed (*P* = 0.087) from the community structure of the digesta in the distal colon (Fig. S4). The administration of 140 mM butyrate altered (*P* = 0.071) the community structure within the colonic digesta but not within the mucosa-associated community in mice without enteritis (Fig. 9). Butyrate affected (*P* ≤ 0.050) the abundance of bacteria within the *Bacteroidetes* (i.e., *Paraprevotelaceae*), *Firmicutes* (i.e., *Bacilli*; *Clostridiaceae*; *Erysipelotrichales*; *Peptostreptococcaceae*) and *Tenericutes* (*RF39*) phyla (Fig. 10). During peak infection, the administration of butyrate showed a trend for reduced (*P* = 0.063) abundance of *Firmicutes*, and, averaged over the two time points, the densities of mucosa-associated *Lachnospiraceae* were reduced (*P* = 0.022) in mice with and without enteritis (Fig. 11 and 12). At late infection, mice inoculated with *C. rodentium* exhibited a lower (*P* = 0.022) abundance of species within the *Bacteroidetes* phylum; however, in mice without enteritis, butyrate supplementation increased (*P* = 0.022) the levels of these bacteria, especially *Parabacteroides* sp. (*P* = 0.023), associated with mucosa averaged over time (Fig. 11 and 12A). An increase (*P* = 0.005) in the abundance of *Gammaproteobacteria* associated with mucosa was also observed during late infection in mice with enteritis that were administered 140 mM butyrate, and in mice without enteritis, butyrate administration increased (*P* = 0.052) the presence of *Bilophila* sp. in association with the mucosa (Fig. 11). Within digesta, butyrate administration altered bacterial communities during late infection (Fig. 11). For example, mice without enteritis that were administered 140 mM butyrate displayed a depletion (*P* = 0.025) of the abundance of *Firmicutes* species, namely, *Clostridiales* (*P* = 0.029) species, and showed a trend of reduced (*P* = 0.067) *Ruminococcaceae* species abundance during late infection. In contrast, mice with enteritis exhibited a higher abundance of *Firmicutes* (*P* = 0.025), *Clostridiales* (*P* = 0.029), and *Lachnospiraceae* species (*P* = 0.014) than mice with enteritis not administered butyrate at late infection (Fig. 11). In mice without enteritis, *Parabacteroides* spp. increased (*P* = 0.023) in average abundance over time with butyrate supplementation (Fig. 12). Although significant differences between the treatments were not evident regarding the abundance of mucus-associated species, butyrate administration caused a trend of reduced *Akkermansia muciniphila* abundance within the digesta collected from the distal colon in mice with and without enteritis (Fig. 12B). In contrast, butyrate administration was associated with a general trend of increased abundance of *A. muciniphila* associated with mucosa (Fig. 12A). Similarly, in mice without enteritis, we observed a trend of increased abundance of the mucus-dwelling bacterium *Mucispirillum schaedleri* in the mucosa-associated community (Fig. 12A). However, a trend of lowered abundance was observed in the digesta within the distal colon, and *M. schaedleri* abundance steadily increased with butyrate administration in mice with enteritis (Fig. 12B). Overall, butyrate administration decreased the abundance of bacteria within digesta and associated with mucosa. However, in digesta collected from the distal colon, butyrate effectively increased the abundance of members of the *Lachnospiraceae* family during infection.

10.1128/mSphere.00243-17.4 FIG S4  Principal-coordinate analysis of bacterial communities within digesta and associated with mucosa in the distal colon from mice inoculated with PBS via gavage, and rectally administered PBS (BU0) or 140 mM butyrate (BU140). Ellipsoids show clustering of communities by sample type. Download FIG S4, PDF file, 0.1 MB. © Crown copyright 2017. 2017 Crown This content is distributed under the terms of the Creative Commons Attribution 4.0 International license.

### Fluorescent *in situ* hybridization (FISH) visualization displayed a high abundance of *Proteobacteria* in butyrate-supplemented tissue.

**FIG 13:**
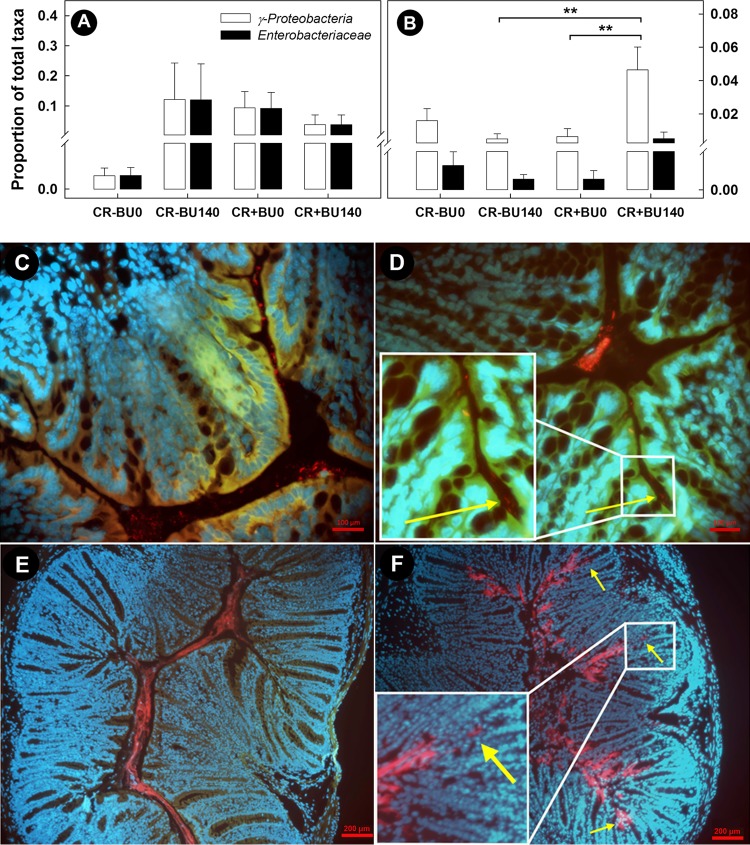
Localization of *Gammaproteobacteria* and evidence of *Proteobacteria* migration into the crypts within the distal colon of mice inoculated with *C. rodentium* (CR+) and rectally administered PBS (BU0) or butyrate at a concentration of 140 mM (BU140). (A) Abundance of *Enterobacteriaceae* and *Gammaproteobacteria* associated with the mucosa on day 14 postinoculation (p.i.) in mice inoculated with PBS and *C. rodentium* (B) Abundance of *Enterobacteriaceae* and *Gammaproteobacteria* associated with mucosa on day 21 p.i. in mice inoculated with PBS and *C. rodentium*. Vertical lines associated with histogram bars represent standard errors of the means (*n* = 4). **, *P* ≤ 0.010 (for treatments linked by the horizontal lines). (C to F) Fluorescence micrographs of distal colonic tissue hybridized with a *Gammaproteobacteria* probe (red). (C) CR+ BU− at day 14 p.i. (bar, 200 µm). (D) CR+ BU+ at day 14 p.i. (bar, 200 µm). (E) CR+ BU− at day 21 p.i. (bar, 100 µm). (F) CR+ BU+ at day 21 p.i. (bar, 200 µm). The arrows indicate subluminal *Proteobacteria*.

To confirm the bacterial community sequencing results, we collected representative tissue and stained for specific groups of bacteria. High densities of *Gammaproteobacteria* and *Enterobacteriaceae* were associated with the mucosa in the distal colon of mice infected with *C. rodentium* compared to mice without enteritis, and high densities of *Gammaproteobacteria* were observed during late infection in mice with enteritis and administered 140 mM butyrate compared to those administered 0 mM butyrate (Fig. 13A and B). It is noteworthy that among the mice with enteritis, higher (*P* = 0.036) densities of mucosa-associated *Pseudomonas* spp. were also observed in digesta from the distal colon of mice administered butyrate than in those not given butyrate (Fig. S5). *Proteobacteria* were more often observed within intestinal colonic crypts in mice rectally administered butyrate at a concentration of 140 mM (Fig. 13D and F) than in mice not administered butyrate (Fig. 13C and E). Butyrate supplementation at a concentration of 140 mM increased the total abundance of *Gammaproteobacteria*, especially in mice infected with *C. rodentium*.

10.1128/mSphere.00243-17.5 FIG S5  Proportion of total sequences within the *Pseudomonadales* order within digesta collected from the distal colon. Vertical lines associated with histogram bars represent standard errors of the means (*n* = 8). *, *P* ≤ 0.050 (for treatments linked by the horizontal lines). Download FIG S5, PDF file, 0.1 MB. © Crown copyright 2017. 2017 Crown This content is distributed under the terms of the Creative Commons Attribution 4.0 International license.

## DISCUSSION

Food consumption and weight gain are characteristics of normal intestinal function and adequate host health, and we observed that infection with *C. rodentium* caused a reduction in food consumption and weight gain, which has been previously reported (34, 35). We also observed that rectal administration of high concentrations of butyrate resulted in an increase in food consumption and weight gain in infected mice, indicating that butyrate plays an important role in improving feeding behavior and enhancing growth during periods of active enteritis. The concentrations of butyrate administered via enema in the current study were higher than would be expected to be generated by bacterial fermentation in both healthy and inflamed murine intestines (36). In rodents, butyrate within the intestine stimulates gluconeogenesis, and high glucose levels within blood activate brain stimuli that promote the metabolism of glucose, improve feed intake, and subsequently promote growth (37, 38). Thus, it is possible that butyrate administered to mice challenged with *C. rodentium* ameliorates bacterially induced colitis by enhancing intestinal gluconeogenesis.

Infection with *C. rodentium* resulted in a strong activation of Th1- and Th17-associated cytokines. These responses are necessary for pathogen clearance (34, 39). Similarly to other studies, we observed elevated levels of epithelial cell hyperplasia in addition to increased expression of *Tnfα*, *Il1β*, *Il17A*, and *Il22* in the distal colon of *C. rodentium*-infected mice (34, 40). In the current study, butyrate treatment further increased expression of cytokines involved in the Th17 and Th1 immune response, which potentially attenuated the infection and promoted epithelial cell restoration (34, 41). This is underscored by the prominent increase in the expression of *Ifnγ*-, *Tnfα*-, *Il1β*-, and Th17-associated cytokines, which are generally used as indicators of active intestinal inflammation (39, 42). Collectively, coordinated Th17 and Th1 immune responses are critical for the clearance of *C. rodentium* (34), and butyrate further stimulated the expression of cytokines involved in resolving the infection (43). It is noteworthy that although the hallmarks of intestinal inflammation following *C. rodentium* challenge were observed, the progression of disease was delayed (44). The strain of *C. rodentium* that was used in the current study was modified to include a chromosomal green fluorescent protein (GFP) insertion, and it is likely that the presence of the insertion altered the bacterial pathogenicity (45).

Expression of the cytokines *Il17A* and *Il17F* is important in the clearance of *C. rodentium* infection, and it has been proposed that these cytokines facilitate the induction of antimicrobial peptides to reduce inflammation (46). Specifically, Th17 cytokines, including IL17 and IL22, have been shown to regulate anti- and proinflammatory and anti- and proapoptotic activities in epithelium cells within inflamed tissue (47–49). These cytokines can also increase production of regulatory proteins such as REGIIIγ during periods of intestinal inflammation (50). We observed that gene expression associated with the intestinal antimicrobial C-type lectin REGIIIγ, an important protein involved in interactions between intestinal bacteria and the epithelium and subsequent tissue repair (51, 52), was increased in mice with enteritis supplemented with butyrate compared to mice with enteritis that were not administered butyrate.

Regulatory T-cell responses are important for modulating and resolving inflammatory responses within the intestine. These responses in association with the stimulation of mucus secretion at the epithelial surface provide an effective strategy to reduce tissue injury following challenges with bacterial pathogens (53, 54). Previous studies have shown that the presence of butyrate increases CD4^+^ *Foxp3* expression from T cells within the colonic mucosa, leading to the mitigation of intestinal injury and promoting inflammatory quiescence (55, 56). Similarly, our findings demonstrated that administration of butyrate at low and high concentrations increased expression of *Tgfβ* and *Il10* in mice with and without enteritis. The maintenance of the physical mucus barrier is important in providing protection to the intestinal epithelium and maintaining intestinal homeostasis (44, 57), and we observed that butyrate administration to mice increased *Muc2* gene expression and deposition of mucus at the epithelial layer. The presence of SCFAs within the intestine has the potential to upregulate mucin secretion (58), and butyrate in particular has been linked to increases in *Muc2* gene expression in cell lines and within the murine colon (6, 10, 59). In contrast, data in support of the ability of butyrate to increase mucus secretion *in vivo* are inconsistent (12, 54, 60). Although our research indicates a relationship between increased mucus production and the rectal administration of butyrate, the amount of butyrate measured in fecal samples was not conspicuously altered in mice administered butyrate relative to those administered buffer rectally. A possible explanation is that butyrate was rapidly absorbed by colonocytes but was less rapidly absorbed in mice with enteritis (3, 11), which was exemplified in our alcian blue–periodic acid-Schiff (AB-PAS)-stained sections.

The maintenance of a protective epithelial barrier is a complex process and includes other proteins involved in the production and secretion of mucus and in enterocyte development and turnover. As an example, accessory proteins such as TFF3 and RELMβ are involved in maintaining epithelial cell homeostasis and regeneration (61), and we observed increased expression of genes encoding both proteins following treatment with butyrate. TFF3 is a trefoil peptide secreted from intestinal goblet cells; it increases mucin viscosity to provide further protection to the mucosa and can enhance epithelial repair in the small and large intestines (62, 63). RELMβ is primarily secreted from goblet cells and is mainly found in localized foci of inflammation in mice and humans (64). The functions of RELMβ are poorly understood; however, it has been associated with colonic immunomodulatory function and contributes to intestinal mucus secretion in mice (65–67). We showed that butyrate administration in mice with enteritis contributed to increased expression of *Relmβ*, *Tff3*, and *Muc2*. Thus, through the modulation of proteins associated with mucus secretion and integrity and epithelial cell repair and restitution, butyrate affects the regulation of mucus-associated proteins during periods of intestinal inflammation.

We observed a general decrease in *Firmicutes* abundance in inflamed intestines in the presence and absence of 140 mM butyrate. Shifts from *Bacteroidetes*-dominant communities to *Firmicutes*-dominant communities in inflamed intestines have been reported previously; however, the evidence is conflicting on whether higher densities of either *Bacteroidetes* or *Firmicutes* correlate with negative or beneficial effects on intestinal health. Our results are in line with those reported by Schwab et al. (31), who observed that chemically induced injury to the colon increased the abundance of bacteria belonging to the *Bacteroidales* order and to *Enterobacteriales* and *Deferribacteres*. Similarly, we found that mice challenged with *C. rodentium* exhibited increases in the abundance of *Deferribacteres* and *Proteobacteria* species, and these changes were similar to the observations of Hoffmann et al. (68). Finally, we also observed a butyrate-dependent increase in levels of *Lachnospiraceae* in digesta within the distal colon, suggesting that butyrate was associated with an increase in the abundance of this group of bacteria. Importantly, *Lachnospiraceae* bacteria produce butyrate in the mammalian intestine (69) and our findings suggest that under conditions of inflammation, these bacteria may increase the amount of butyrate within the colon and subsequently mitigate inflammation (11). Furthermore, as our results suggest that butyrate is a putative inducer of mucus-associated bacteria, our data may signify an increase in the availability of mucus for microbial metabolism. Our data also showed trends of increased abundance of mucolytic *A. muciniphila* bacteria at the mucosal surface in response to butyrate treatment (70). Increases in the abundance of *A. muciniphila* during periods of dietary change and intestinal inflammation have also been linked to increased expression of host mucus and immune modulating genes (71, 72), suggesting that this bacterium induces both production and utilization of mucin during episodes of colitis. Collectively, our findings provide evidence that during infection, butyrate modulates the bacterial community in the inflamed distal colon to promote the growth of butyrogenic bacterial species, which also stimulate the butyrate-driven production of mucus by the host.

Although butyrate is an SCFA that has been implicated in improved barrier function, promotion of intestinal epithelial cell growth and repair, and enhanced host mucin secretion (6, 10, 23), we observed that administration of butyrate directly to the colon mildly exacerbated infection in mice with enteritis. In this regard, mice with enteritis that were treated with a low concentration of butyrate tended to have modestly higher histological scores during peak and late infection that had significantly subsided by the time of infection clearance. Histological scores also tended to be the highest in mice treated with butyrate at 80 mM compared to those treated with butyrate at a concentration of 140 mM, suggesting that butyrate administration works at a particular threshold and is most effective at reducing inflammation under conditions of administration to mice at high concentrations. As indicated above, we observed that butyrate-treated mice challenged with *C. rodentium* showed differing increases in gene expression of cytokines involved in the clearance of infection (*Il17A*, *Tnfα*), the reduction of mucosal inflammation (*Tgfβ*, *Il10*), and the improvement of host barrier function (*Muc2*, *Relmβ*, *Tff3*). These findings suggest that other processes that stimulate various cytokine pathways may also contribute to modestly heightened levels of tissue inflammation. As an example, in the presence of *C. rodentium*, butyrate can cause activation of the promoter of the locus of enterocyte effacement (LEE) operon and expression of *ler* genes necessary for the expression of virulence factors that can exacerbate tissue inflammation (73, 74). Moreover, morphological transformations in the mucosa following *C. rodentium* infection have been associated with a hyperreactive epithelial reparative response that induces changes such as marked epithelium hyperplasia with increased production of undifferentiated enterocytes (43). This could also potentially increase inflammation scores. In the current study, enhanced expression of epithelial cell-regenerating proteins such as MYD88, TFF3, and RELMβ in butyrate-treated mice likely further elevated the inflammation scores (i.e., as a consequence of increased epithelial hyperplasia, crypt height, and mitotic activity) for *C. rodentium*-challenged mice, masking some of the anti-inflammatory effects and accentuating proinflammatory tissue responses. Finally, we observed an increase in the abundance of the mucus-degrading bacterium *A. muciniphila* at the mucosal surface in butyrate-treated mice that was not observed in digesta in the distal colon. As indicated previously, *A. muciniphila* is known to colonize the intestinal mucus layer, and its presence has been associated with increased epithelial cell turnover, increased biosynthesis of epithelial cell components, and enhanced mucin production (70). Therefore, it is likely that the presence of *A. muciniphila* increased epithelial growth (i.e., hyperplasia turnover of colonocytes to increase total histological inflammation scores), and this warrants further investigation.

In conclusion, our findings provide evidence that, in a dose-dependent manner, butyrate administration can improve the weight gain of infected mice, enhance clearance of the infection, reduce inflammation through altered cytokine expression, and enhance tissue repair and mucus secretion. Moreover, butyrate treatment also affected the abundance of bacterial populations in both noninflamed and inflamed intestines. Notably, this investigation provided foundational information that can be used to determine the effects of prebiotics and other functional foods on the production of butyrate by enteric bacteria and their impact on intestinal health and host well-being.

## MATERIALS AND METHODS

### Experimental design.

The experiment was arranged as a completely randomized design with four levels of butyrate concentration (0 mM, 80 mM, 100 mM, and 140 mM), two levels of immunological stress (with *C. rodentium* and without *C. rodentium*), and three levels of time postinoculation (p.i.) (14, 21, and 28 days p.i.). Each replicate included 24 mice, and four replicates were performed on separate occasions (96 animals in total).

### Ethics statement.

The study was carried out in strict accordance with the recommendations specified in the Canadian Council on Animal Care Guidelines. The project was reviewed and approved by the Lethbridge Research and Development Center (LRDC) Animal Care Committee (Animal Use Protocol Review 1322) and the LRDC Biosafety and Biosecurity Committee before commencement of the research.

### Mouse maintenance.

Specific pathogen-free (SPF) C57BL/6J female mice were obtained from Charles River Laboratories, Inc. (Montreal, Quebec, Canada) at 3 weeks of age. For each replicate, mice were housed in groups with six mice per cage upon arrival and were given 10 days to adapt to the animal facility environment under conditions of a 10-h/14-h dark/light cycle. After the adaptation period, mice were transferred to individually ventilated cages (one mouse per cage) operated in containment mode. Mice were provided a low-fiber diet (AIN-93G 103455GI; Dyets Inc., Bethlehem, PA) and were permitted to eat and drink *ad libitum*. Sterile shredded paper was provided for bedding. The health status of each mouse was monitored daily using a quantitative scoring system (75). Cages (including bedding, food, and water) were replaced weekly. Initial body weights were taken a day before the initial enema and gavage inoculations and again at the time of euthanization. Overall weight gain and feed consumption were measured.

### Butyrate administration.

A stock solution of butyric acid (Sigma-Aldrich, Oakville, Ontario, Canada) (>99%; molecular mass, 88.11 g/mol; 100 ml) was diluted with 1× phosphate-buffered saline (PBS) (Sigma-Aldrich) (0.01 M NaH_2_PO_4_, 0.04 M Na_2_HPO_4_, 0.07 M NaCl; pH 7.4) to attain final concentrations of 80 mM, 100 mM, and 140 mM butyrate; the pH was adjusted to 7.4 ± 0.2 with 10 M sodium hydroxide. The butyrate solution was prepared the day prior to administration and was stored at 4°C until use. Solutions were warmed to room temperature (RT) for 30 min before administration. Phosphate-buffered saline (PBS) served as the butyrate control treatment. Butyrate treatments were administered via enemas (300 µl) at 2-day intervals throughout the experimental period. For administration of enemas, mice were inverted at a 45° angle, and a 22-gauge-by-2.5-cm-long gavage needle with a 1.25-mm ball tip was gently inserted into the colon, the liquid was slowly injected, and mice were maintained in an inverted position for 30 s after administration of the enema. Animals were monitored for discomfort/pain for 4 h after the enemas were administered.

### *Citrobacter rodentium* inoculation.

Green fluorescent protein-labeled *C. rodentium* DBS100 (ATCC 51459) was used to incite acute inflammation. The bacterium was grown aerobically on lysogeny broth agar (LA) with 30 µg/ml chloramphenicol at 37°C for 24 h. To differentiate GFP-labeled *C. rodentium* from nonlabeled *C. rodentium*, a chloramphenicol resistance gene was incorporated into the genome, which required chloramphenicol to be used for growth and isolation techniques. Biomass was removed from the surface of the agar and transferred into sterile lysogeny broth (LB) containing 15 µg/ml chloramphenicol (Sigma-Aldrich). Cultures were maintained for 2 h at 37°C at 100 rpm until an optical density at 600 nm (OD_600_) of >0.1 was obtained. Cultures were centrifuged at 2,256 × *g* for 15 min, supernatants were removed, and *C. rodentium* cells were resuspended in 3.0 ml PBS. To confirm densities of viable cells, inoculum was diluted in a 10-fold dilution series and 100 µl of each dilution was spread in duplicate onto LA. Cultures were incubated at 37°C, and the number of *C. rodentium* colonies was counted at the dilution yielding 30 to 300 CFU after 24 h. Cell densities were adjusted to 3 × 10^9^ CFU/ml with PBS. For each replicate, 12 mice were administered *C. rodentium* cells and PBS (100 µl) or PBS alone (100 µl) via gavage on two consecutive days using a 22-gauge-by-2.5-cm-long gavage needle with a 1.25-mm ball tip.

### Quantification of *C. rodentium* in feces.

Fecal samples from mice were collected at ∼7-day intervals and homogenized in 1.0 ml LB, the homogenate was diluted in a 10-fold dilution series, and 100-µl aliquots of each dilution were spread in duplicate on MacConkey agar (Becton, Dickinson and Company, Mississauga, Ontario, Canada) containing 15 µg/ml chloramphenicol (Sigma-Aldrich). Cultures were incubated at 37°C for 24 h, and colonies of *C. rodentium* were enumerated at the dilution yielding 30 to 300 CFU. To confirm the identity of *C. rodentium*, colony PCR was performed on arbitrarily selected colonies. The EspB protein is responsible for *C. rodentium* attachment to host membranes to cause infection, and this effector molecule was chosen as a target gene for identification (76). Primers specific for the *espB* gene (CrodF [5′-GCTTCTGCGAAGTCTGTCAA-3′] and CrodR [5′-CAGTAAAGCGACTTAACAGATT-3′]) were used to confirm the identity of *C. rodentium* isolates (36). PCR conditions commenced with one cycle of 15 min at 95°C, followed by 35 cycles of 45 s at 95°C, 1 min at 57°C, and 1 min at 72°C and a final cycle of 5 min at 72°C. The amplicon was 270 bp in size.

Densities of *C. rodentium* were also enumerated in feces on days 14, 21, and 28 p.i. using SYBR green quantitative PCR (qPCR). Genomic DNA from feces (200 mg ± 5 mg) was extracted using a QIAamp DNA stool minikit (Qiagen Inc.) according to the manufacturer’s recommendation. The primers targeting the *espB* gene described above were used. PCR was conducted using an Mx 3005p thermocycler (Agilent Technologies Canada Inc., Mississauga, Ontario, Canada). The conditions for amplification were 1 cycle at 95°C for 15 min followed by 40 cycles of 15 s at 94°C, 30 s at 57°C, and 30 s at 72°C for data acquisition. Melt curve analysis was conducted over a range of 55 to 95°C, with increments set at 0.5°C (80 cycles). A linear equation established from genomic DNA extracted from *C. rodentium* bacteria of known densities was used to interpolate the numbers of copies present in the unknown samples. In all instances, each sample was run twice (i.e., two subsamples), and the mean value was used.

### Animal euthanization and intestinal sample collection.

On days 14, 21, and 28 p.i., one randomly selected mouse from each treatment was anesthetized with isoflurane followed by euthanasia by cervical dislocation under anesthesia. Immediately after death, a mid-line laparotomy was used to exteriorize the intestine, and a gross pathological assessment of the intestine was completed. The colon was longitudinally incised, and digesta was collected and stored at −20°C for analysis of SCFAs. Sections of distal colon (∼4 mm in length) were weighed and placed at −20°C for DNA analyses and in RNAlater (Qiagen, Toronto, Ontario, Canada) at −20°C for mRNA extraction. Tissue from the distal colon was also collected for histopathologic and mucus analyses, as well as for fluorescent *in situ* hybridization (FISH).

### Histopathology.

Harvested colonic tissue was fixed in Surgipath 10% neutral buffered formalin (Leica Biosystems, Concord, Ontario, Canada) for 24 h. Formalin-fixed tissues were dehydrated in ethanol and placed in Histo-Clear (Diamed Lab Supplies, Mississauga, Ontario, Canada) prior to embedding in paraffin at 60°C. Sections (5 µm) were deparaffinized with xylene and stained with hematoxylin and eosin (H&E). Tissues were scored for mucosal damage by an experienced veterinary pathologist blind to the treatments using an established scoring guide (34) that ranked common characteristics of mucosal damage at scores from 0 to 4, with 4 representing pronounced damage and 0 representing minimal to no damage (see Table S1 in the supplemental material). Sections were also scored (0 to 3 or 4) for epithelial cell wall hyperplasia based on a mild to severe increase in the number of cells found within crypt columns; for crypt height based on minimal to maximal increases in height; for epithelium cell injury (noting the degree of focal erosions and cell shedding); for the degree of inflammation based on the number of neutrophils and mononuclear cells present in the lamina propria; for goblet cell depletion based on the number of goblet cells and mucin droplet size; and for the degree of mitotic activity based on how much of the epithelial cell displayed increased activity (Table S1).

10.1128/mSphere.00243-17.6 TABLE S1  Description of the histological scoring criteria applied to cross sections from the murine distal colon. Download TABLE S1, PDF file, 0.1 MB. © Crown copyright 2017. 2017 Crown This content is distributed under the terms of the Creative Commons Attribution 4.0 International license.

### Short-chain fatty acid analysis.

To quantify SCFA levels, fecal pellets were collected and stored on ice until they were weighed (within 30 min of collection). After the weight was calculated, samples were homogenized in PBS at a 1:9 (wt/vol) ratio. Meta-phosphoric acid (Sigma-Aldrich) was added to the homogenate at a 1:4 (vol/vol) ratio, and the reaction mixture was incubated at RT for 30 min. Samples were then centrifuged for 75 min at 16,100 × *g*, and the supernatants were collected and stored at −20°C. Acetate, butyrate, and propionate concentrations were quantified with a gas chromatograph (model 6890 N with 7683 series injector; Agilent Technologies, Mississauga, Ontario, Canada) according to an established protocol (77, 78).

### Characterization of digesta- and mucosa-associated bacterial communities.

Mucosa-associated bacterial genomic DNA was extracted from distal colonic samples using a DNeasy blood and tissue extraction kit (Qiagen Inc.). Genomic DNA was also extracted from the distal colonic digesta using a QIAamp Fast DNA stool extraction kit (Qiagen Inc.). Extracted DNA was processed using an Illumina protocol for creating 16S rRNA gene metagenomic sequencing libraries (79). Extracted DNA was normalized to 5 ng/µl in 10 mM Tris (pH 8.5). Following this, 2.5 µl of purified DNA was PCR amplified with 5 µl of each amplicon primer, spanning the V3 and V4 regions of the 16S rRNA gene (F [5′-TCGTCGGCAGCGTCAGATGTGTATAAGAGACAGCCTACGGGNGGCWGCAG-3′] and R [5′-GTCTCGTGGGCTCGGAGATGTGTATAAGAGACAGGACTACHVGGGTATCTAATCC-3′]), and 12.5 µl of 2× Kapa HotStart Ready mix (Kapa Biosystems, Inc., Wilmington, MA) for a final volume of 25 µl. The resulting 550-bp product underwent a PCR cleanup using AMPure XP beads (Beckman Coulter Canada Inc., Mississauga, Ontario, Canada) on a magnetic stand to isolate the DNA, and the product was washed with 80% ethanol and eluted with 10 mM Tris (pH 8.5). An indexing PCR was used to add forward and reverse indices to each sample. Conditions included 5 µl of DNA, 5 µl of each index primer (specific nonrepeating pair per sample), and 25 µl of 2× Kapa HiFi HotStart Ready mix and nuclease-free water (Qiagen Inc.) added to reach a final volume of 50 µl per sample. A final PCR cleanup was performed on the 630-bp product. Indexed DNA libraries were quantified and normalized to 4 nM with 10 mM Tris (pH 8.5), and 5 µl of each normalized library was pooled into one sample for sequencing using a MiSeq system (Illumina, San Diego, CA). A PhiX control was run in parallel with the normalized DNA libraries, and both were denatured and diluted to 4 pM prior to loading onto the MiSeq cartridge.

Forward reads were assembled using the Quantitative Insights Into Microbial Ecology (QIIME; version 1.8.0) software package (80), resulting in a total of 13,125,994 sequences. Barcodes were extracted from each sample FASTQ file, and each was joined with its corresponding forward read. Libraries were split according to barcode, and sequences were filtered to include only those sequences with a base-calling-accuracy Phred value (Q) of 20 or greater, indicating the probability of 1 in 100 base calls being incorrect. Sequence reads were filtered to exclude those with more than three consecutive low-quality base calls, those with less than 75% of the read length containing consecutive high-quality base calls, and/or those corresponding to barcode having more than 1.5 errors present. These sequences (*n* = 5,390,077) were then subjected to chimera checking using USEARCH 6.1 software, and the resulting chimeras were filtered out prior to picking of operational taxonomic units (OTU) from the Greengene reference database. In total, 371,065 OTUs were identified using a 97% similarity parameter, and the most common sequence was used to define the groups of similar OTUs. OTUs were then aligned to the Ribosomal Database project (RDP) with a classifier value of 0.5 (sequences having at least 50% similarity to reference database sequences) using the NAST algorithm (81). Taxonomies were assigned to each sequence cluster using UCLUST (82) and classified using the Greengenes reference database (83). An OTU table was produced, and data from all samples were rarified such that 3,450 OTUs were randomly chosen and compared between samples for analysis (the number of OTUs per biological sample ranged from 3,450 to 100,000). Diversity among species (β-diversity) was examined using Bray-Curtis analysis and weighted and unweighted UniFrac analyses (84).

### Visualization of intestinal bacteria.

The presence and localization of bacteria within the colon were determined using FISH. Preparation of colonic samples for FISH was performed using the method described below for AB-PAS staining. Tissue sections were circled with a hydrophobic pen, and sections were incubated in the dark overnight at 37°C with either the Alexa Fluor 555-conjugated total bacterial probe EUB338 (Life Technologies, Inc., Burlington, Ontario, Canada) (5′-GCTGCCTCCCGTAGGAGT-3′) or the Alexa Fluor 555-conjugated *Gammaproteobacteria* probe Gam42a (Life Technologies, Inc.) (5′-GCCTTCCCACATCGTTT-3′). Probes were stored in 0.25 µg/µl stocks and diluted (1:100) with hybridization buffer (0.9 M NaCl, 0.1 M Tris [pH 7.2], 30% formamide, 0.1% SDS) prior to addition of the solutions to the sections. The sections were stored in a dark humidifying box. After incubation with the fluorescent probe, sections were washed with hybridization buffer in the dark for 15 min and then with wash buffer (0.9 M NaCl, 0.1 M Tris [pH 7.2]). Sections were mounted with ProLong Gold Antifade solution with DAPI (4′,6-diamidino-2-phenylindole) (44) and analyzed with a Zeiss Axioskop II Plus microscope (Carl Zeiss Canada, Ltd., North York, Ontario, Canada) using Zen2 (Blue edition) core imaging software.

### Quantification of gene expression.

Gene expression (Table S2) was analyzed for cytokine profiles from total RNA that was extracted using an RNeasy minikit (Qiagen Inc.). The concentration and quality of the total RNA extracted were analyzed using an RNA 600 Nano Chip and a 2100 Bioanalyzer (Agilent Technologies). Using 1,000 ng of total RNA, reverse transcription was performed using a QuantiTect reverse transcription kit (Qiagen Inc.). The reference genes used to normalize the measured threshold cycle (*C*_*T*_) values were *Ppia*, *Hprt*, and *GusB*. Quantitech SYBR green Mastermix (Qiagen Inc.) was used as an indicator of double-stranded DNA and product amplification. Individual PCRs consisted of 1 µl of cDNA; 3 µl of nuclease-free water (Qiagen Inc.); 0.5 µl of 10 µM forward primer; 0.5 µl of 10 µM reverse primer; and 5 µl SYBR green (Table S2). Reactions were run in triplicate per cDNA sample. Quantitative PCRs were run on a 384-well ABI 7900HT qPCR thermocycler (Life Technologies, Inc.), with an activation step of 95°C for 15 min and 40 cycles of 94°C for 15 s, 58°C for 30 s, and 72°C for 30 s, followed by melt curve analysis. Normalized gene expression data were calculated using qbasePLUS (Biogazelle, Zwijnaarde, Belgium) on the basis of geNorm and qBase quantification models (85, 86).

10.1128/mSphere.00243-17.7 TABLE S2  Targets and primer sequences used to analyze the gene expression of cDNA harvested from murine colonic tissue. Download TABLE S2, PDF file, 1 MB. © Crown copyright 2017. 2017 Crown This content is distributed under the terms of the Creative Commons Attribution 4.0 International license.

### Characterization of mucus.

Colonic tissue samples were collected from the same region of the distal colon from each sampled mouse and were fixed overnight in methacarn (60% methanol, 30% chloroform, 10% glacial acetic acid) (87) prior to dehydration with ethanol and Histo-Clear (Diamed Lab Supplies). Sections (5 µm) were deparaffinized for 5 min on a 60°C heating bed, cleared with xylene, and rehydrated in a decreasing ethanol gradient (100%, 90%, 70%, and 50%) according to a standard protocol (Abcam, Inc., Toronto, Ontario, Canada). To visualize mucus, sections were stained with alcian blue (American MasterTech, Lodi, CA) (pH 2.5) for 30 min, 0.5% periodic acid (American MasterTech, Lodi, CA) for 5 min, and Schiff’s solution (American MasterTech, Lodi, CA) and were stored at 4°C for 15 min (AB-PAS).

### Statistical analyses.

Most statistical analyses were performed using SAS (SAS Institute, Cary, NC). Continuous data were checked for normality and analyzed using the MIXED procedure of SAS (SAS Institute, Cary, NC). Where applicable (i.e., for samples were not independent), collection time was treated as a repeated measure; the appropriate covariance structure was utilized according to the lowest Akaike’s information criterion. In the event of a significant main effect, the least-squares method (LSM) was used to compare treatments within factors. Categorical data (i.e., histopathology results) were analyzed using the GLIMMIX procedure. For bacterial community analyses, both SAS and Primer 7 were used. In Primer 7, PERMANOVA (permutational multivariate analysis of variance) and principal-coordinate analyses (PCoA) were used to determine levels of β-diversity whereas analysis of variance (MIXED procedure) was used with a protected LSM test to determine levels of α-diversity. Differences in the abundances of microbial OTUs were also analyzed using the MIXED procedure with a protected LSM test. For both parametric and nonparametric statistical analyses, *P* values of ≤0.050 were considered to represent statistical significance, whereas *P* values of >0.050 and ≤0.100 were considered to represent a statistically significant trend.
