# Supplementary figures and images for: Butyrate Supplementation at High Concentrations Alters Enteric Bacterial Communities and Reduces Intestinal Inflammation in Mice Infected with Citrobacter rodentium
Source: mSphere. 2017 Aug 23;2(4):e00243-17. doi: 10.1128/mSphere.00243-17 (PMC5566833; doi:10.1128/mSphere.00243-17)

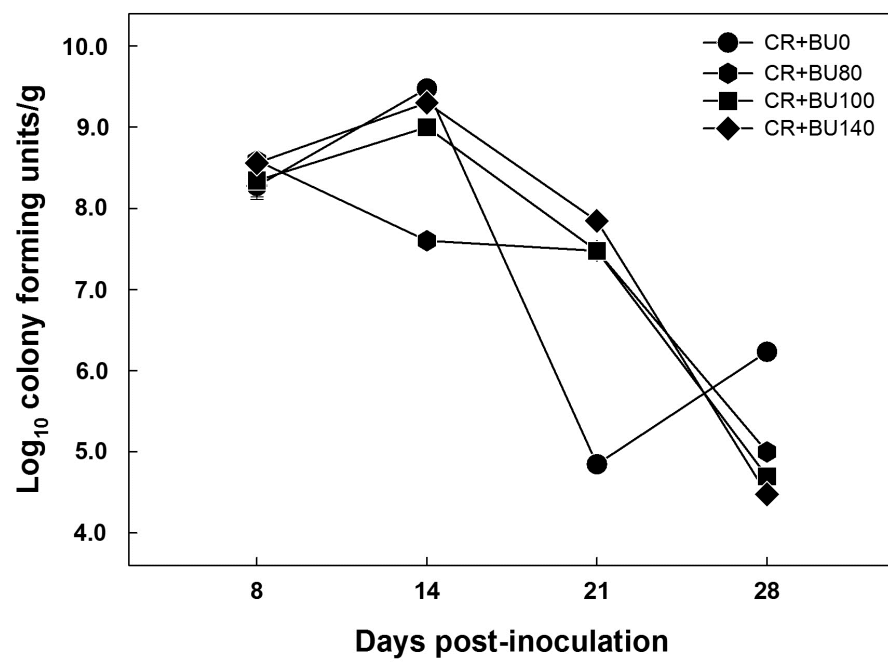

FIG S1

Supplement: FIG S1 [file sph004172345sf1.pdf]

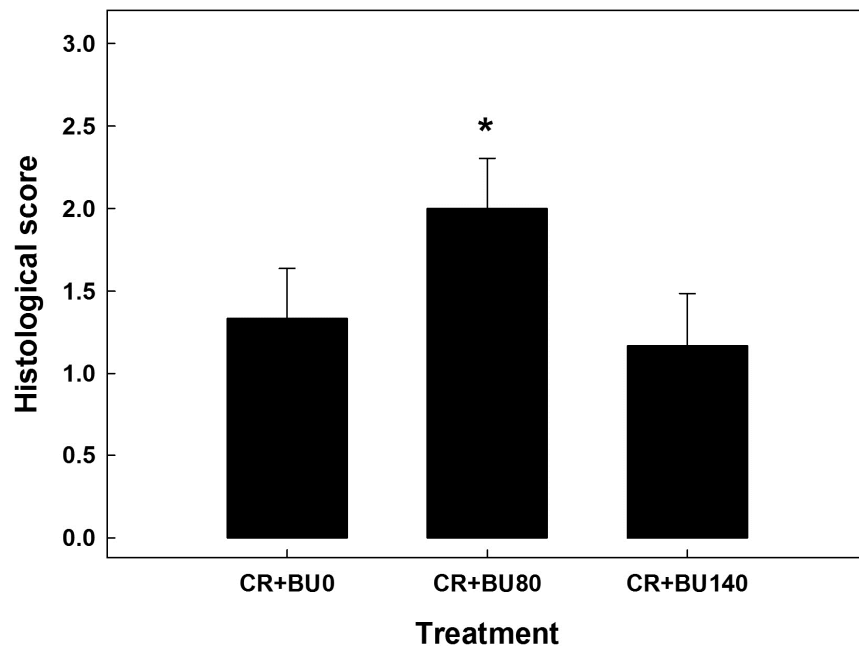

FIG S2

Supplement: FIG S2 [file sph004172345sf2.pdf]

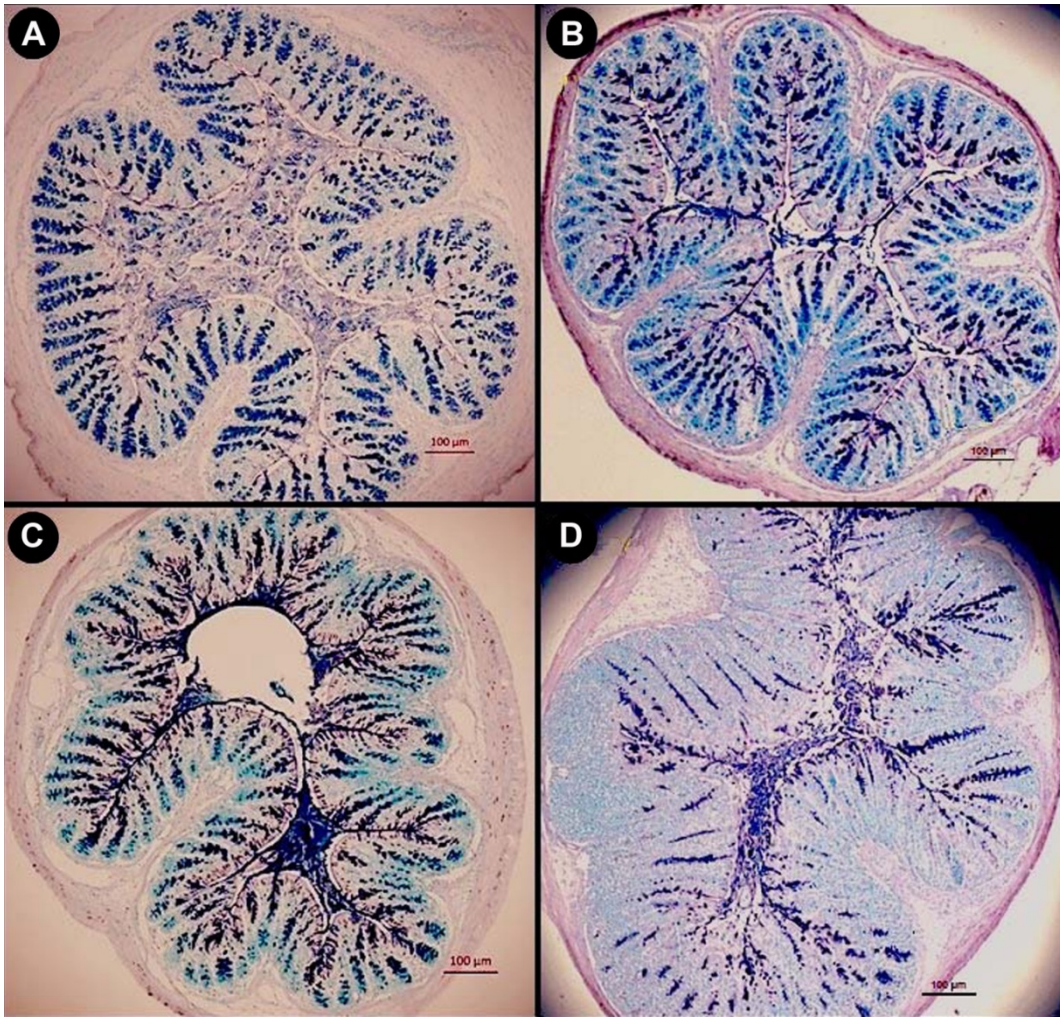

FIG S3

Supplement: FIG S3 [file sph004172345sf3.pdf]

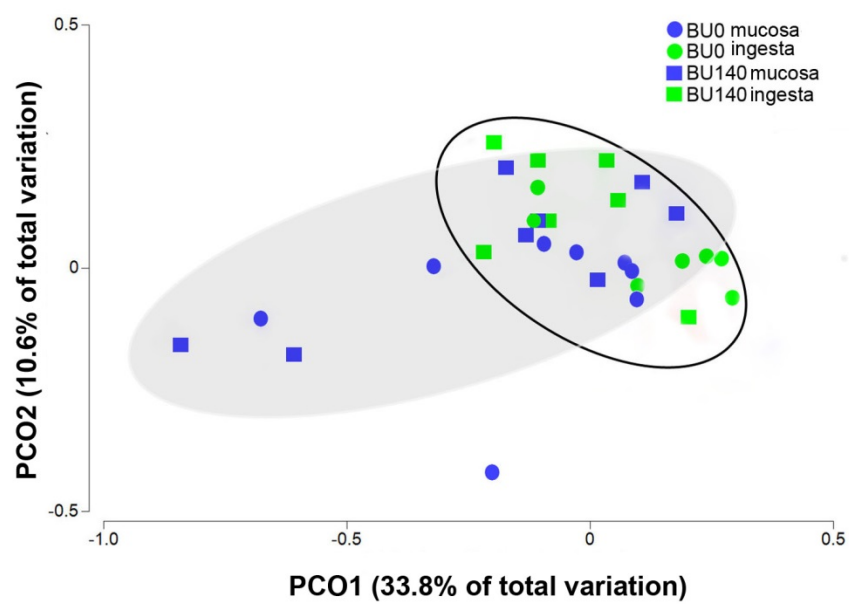

FIG S4

Supplement: FIG S4 [file sph004172345sf4.pdf]

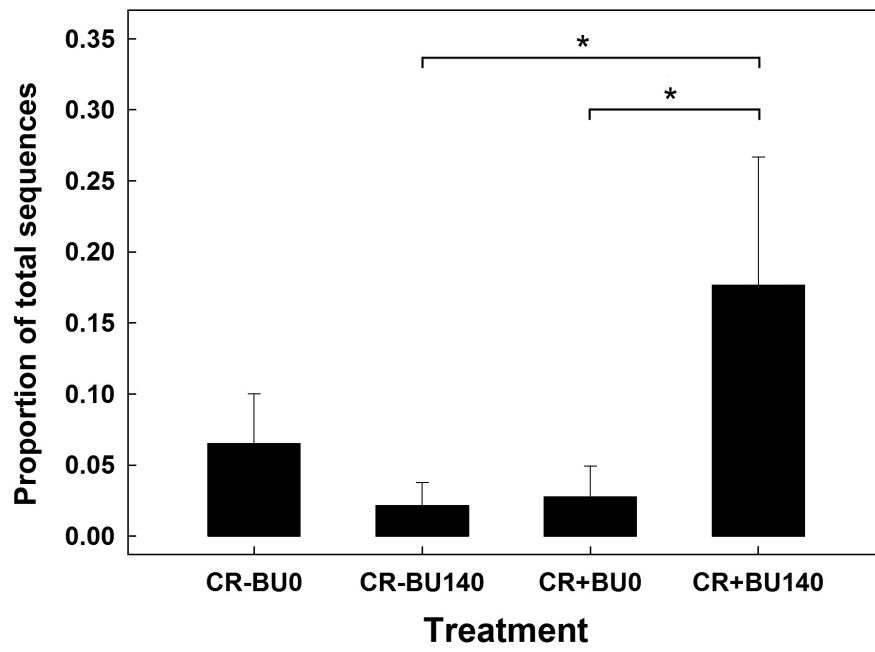

FIG S5

Supplement: FIG S5 [file sph004172345sf5.pdf]
