# Supplementary material for: Butyrate Supplementation at High Concentrations Alters Enteric Bacterial Communities and Reduces Intestinal Inflammation in Mice Infected with Citrobacter rodentium
Source: mSphere. 2017 Aug 23;2(4):e00243-17. doi: 10.1128/mSphere.00243-17 (PMC5566833; doi:10.1128/mSphere.00243-17)
Supplement: TABLE S1 [file sph004172345st6.pdf]

Table S1. Description of the histological scoring criteria applied to cross-sections from the murine distal colon.

Histological Category	0	1	2	3	4
**Epithelial cell wall hyperplasia**	None	Mild increase, mucosal crypt columns 2x the normal amount of cells, focal areas of hyperplasia	Moderate increase, mucosal crypt columns contain 3x the normal amount of cells, crowding of cells in base of crypts	Marked increase, mucosal crypt columns contain 4x the normal amount of cells, crowding of cells in base of crypts	Severe increase, villous distortion, mucosal crypts contain more than 5x the normal amount of cells, focal dysplasia of epithelium surface
**Crypt height**	Normal	Mild increase, 25% increase crypt height	Moderate increase, 50% crypt height	Marked increase, 100% increase crypt height	Severe increase, >100% increase crypt height
**Epithelial cell injury**	None	Superficial; mild <10 surface epithelial cells shedding	Moderate, focal erosions, 11-20 surface epithelial cells shedding	Marked, multi-focal erosions of surface epithelial cells	Severe, multifocal erosions with or without deep crypt necrosis
**Inflammation**	None	Rare numbers of neutrophils and/or mononuclear cells present within the lamina propria	Small numbers of neutrophils and or mononuclear cells present within the lamina propria	Large numbers of neutrophils and/or mononuclear cells within the lamina propria that on occasion focal to multi-focally extend into submucosa, muscularis and serosa	Large numbers of neutrophils and/or mononuclear cells within extensive areas of the lamina propria, submucosa, muscularis and serosa
**Goblet Cell Depletion**	Normal	Low depletion of goblet cells, smaller size of mucin droplets	Evident depletion of goblet cells, marked decrease of the size of mucin droplets	Absent	Not applicable
**Mitotic Activity**	Normal	Mild, small increase in mitotic activity in deep crypt epithelial cells	Moderate, prominent increase in mitotic activity in the basal half of crypt epithelial cells	Marked, prominent increase in mitotic activity that extends the entire length of crypt epithelial cells	Not applicable

The maximal total score for tissue inflammation is 22.
