# Supplementary material for: Butyrate Supplementation at High Concentrations Alters Enteric Bacterial Communities and Reduces Intestinal Inflammation in Mice Infected with Citrobacter rodentium
Source: mSphere. 2017 Aug 23;2(4):e00243-17. doi: 10.1128/mSphere.00243-17 (PMC5566833; doi:10.1128/mSphere.00243-17)
Supplement: TABLE S2 [file sph004172345st7.pdf]

Table S2. Targets and primer sequences used to analyze the gene expression of cDNA harvested from murine colonic tissue.

**Primer**	**Sequence (5' to 3')**
*Tnfα**	F:GATCGGTCCCCAAAGGGATG<br>R: GCTCCTCCACTTGGTGGTTT
*Tgfβ**	F: GTCCAAACTAAGGCTCGCCA<br>R: CATAGTAGTCCGCTTCGGGC
*Reg3γ**	F: GATGCCCCATCTTCACGTAG<br>R: ACAAGGCATAGCAATAGGAGC
*MyD88**	F: ATCGCTGTTCTTGAACCCTC<br>R: CCAAGTACTCGAAGCCCATC
*Muc2**	F: AAAGACCACAACAGGGCCAA<br>R: GGTCCTGGTGGTCTCCAAAG
*Il10**	F: ACAGCCGGGAAGACAATAAC<br>R: GGCAACCCAAGTAACCCTTA
*Il4**	F: AGCAACGAAGAACACCACAG<br>R: TCGAAAAGCCCGAAAGAGTC
*Il17α**	F: GCAGCGATCATCCCTCAAAG<br>R: ACGTGGAACGGTTGAGGTAG
*Il1β**	F: GTGTCTTTCCCGTGGACCTT<br>R: GGAGCCTGTAGTGCAGTTGT
*Il22**	F: TGACACTTGTGCGATCTCTGA<br>R: CTTGCACCGGGTGTTGACG
Infγ (1)	F: ACGGCACAGTCATTGAAAGC<br>R: TCTGGCTCTGCAGGATTTTCA
*Tlr2**	F: GCTCCTGCGAACTCCTATCC<br>R: CAGCAGACTCCAGACACCAG
*Tlr9**	F: CAGTTGCCGACTGGGTGTAT<br>R: GAGTCTTGCGGCTCCCATAG
*Tff3**	F: TCTGGCTAATGCTGTTGGTG<br>R: ATACATTGGCTTGGAGACAGG
Prg3†	F: AGCATAGAAGCTGCGTTGGA<br>R: GATCAGGGGCTGTTTCATGT
*Relmβ**	F: TCTCAGTCGTCAAGAGCCTAA<br>R: AAGCACATCCAGTGACAACC
LtB4r1†	F: AAACCCTGTCCTTTTGATGGC<br>R: AGAACAATGGGCAACAGAGA

†Primer sequences developed from PCR arrays specific for butyrate-induced inflammation.*Primers developed for this study using the NCBI database.

Reference

1. Ramos-Payán R, Aguilar-Medina M, Estrada-Parra S, González-y-Merchand JA, Favila-Castillo L, Monroy-Ostria A, Estrada-Garcia ICE. 2003. Quantification of Cytokine Gene Expression Using an Economical Real-Time Polymerase Chain Reaction Method Based on SYBR® Green I. Scand J Immunol 57:439-445.
